# 

**Published:** 1872-05

**Authors:** 


### CONTENTS:

### ORIGINAL COMMUNICATIONS.

ARTICLE I.—Neuralgia as Pertaining to the Dental Organs. By N. E. Hollace, M.D ....... 1

ARTICLE II.—Nitrous Oxide Gas Experiments. By S. P. Cutler, M.D., D.D.S................ 3

ARTICLE III.—Itinerant Dentistry. By Joseph H. Scales, D.D.S.. ........................... 9

ARTICLE IV.—Second District Dental Society of New York. By J. C. Wyman, Sect'y..... 11

ARTICLE V.—Southern Dental Association. By O. J. Bond, Rec. Sect'y......................... 13

### SELECTED ARTICLES.

ARTICLE VI.—Death from Inhalation of Nitrous Oxide Gas. By W. J Purcell, M.D....... 13

ARTICLE VII.—Treatment of Children's Teeth. By C. E Francis, D.D.S.......................... 17

ARTICLE VIII.—Cancrum Oris. By C. S. Kittredge, M.D........................................... 22

ARTICLE IX.—The Teeth and Mouth of Idiots.................................................... 27

ARTICLE X.—Pluggers. By H. S. Chase, D D.S..................................................... 30

ARTICLE XI.—Removal of Large Fibro-Cystic Tumour of Lower Jaw.

By Christopher Heath, F.R.S.............................. 33

ARTICLE XII.—On the Swallowing of Insoluble Foreign Bodies by Children.

By Jno. H. Packard. M. D ................................. 38

ARTICLE XIII.—A New Method of Hardening Rubber. By Jas. E. McCabe...................... 39

ARTICLE XIV.—Severe Ptyalism from a Dental Operation Without Amalgam

By A. Berry, D.D.S.............................................. 40

ARTICLE XV.—An Interesting Operation. By Mary J. Safford, M.D............................ 41

### MONTHLY SUMMARY.

Danger from the Vapor of Mercury.............................................................. 42
Purification of Carbolic Acid...................................................................... 44
On the Effects of Diseased Meat as Food...................................................... 44

### EDITORIAL DEPARTMENT.

Hypertrophy of the Maxilla........................................................................ 45
Removing Salivary Calculus with Acids.......................................................... 45
Careful Examinations................................................................................ 46
Prophylactic Measures for Decay of the Teeth............................................... 47
The Cumming's Patent................................................................................ 47

### OBITUARY.

Dr Elisha Baker.......................................................................................... 48

